# Cesarean myomectomy: a case report and review of the literature

**DOI:** 10.1186/s13256-021-02785-7

**Published:** 2021-04-24

**Authors:** Priyanka Garg, Romi Bansal

**Affiliations:** 1 Department of Obstetric and Gynecology, All India Institute of Medical Sciences, Bathinda, 151505 India; 2 Department of Obstetric and Gynecology, Adesh institute of Medical Sciences and Research, Bathinda, Punjab India

**Keywords:** Myomectomy, Cesarean, Postpartum hemorrhage, Pregnancy, Fibroid

## Abstract

**Background:**

Routine myomectomy at the time of cesarean section has been condemned in the past due to fear of uncontrolled hemorrhage and peripartum hysterectomy. It is still a topic of debate worldwide. However, in recent years, many case studies of cesarean myomectomy have been published validating its safety without any significant complications.

**Case presentation:**

We describe the case of a 27-year-old gravida 2 para 1 live birth 1 North Indian woman with one previous lower segment caesarean section (LSCS) at 35 weeks with labor pains and scar tenderness. Her recent ultrasound (USG) report suggested a single live intrauterine pregnancy with an intramural fibroid of 8.6 × 6.5 cm located in the left anterolateral wall of the lower uterine segment. The patient was taken up for emergency cesarean section along with successful removal of the myoma, which was bulging into the incision line, causing difficulty in closure of the uterine wound. Prophylactically, oxytocin infusion, bilateral ligation of uterine arteries, and injection vasopressin (diluted) was administered to decrease the blood loss. The patient was discharged after 7 days without any complications.

**Conclusions:**

Routine myomectomy at the time of cesarean section is not a standard procedure and is not accepted worldwide. However, it may be considered a safe option in carefully selected cases in the hands of an experienced obstetrician with appropriate hemostatic technique. Large multicenter randomized controlled trials should be conducted to evaluate the best practice guidelines for cesarean myomectomy.

## Background

Leiomyomas are the most common benign tumors of the reproductive tract in women of childbearing age. Their exact incidence in pregnancy is hard to estimate. However, literature reports a prevalence of 2–4% [1]. The incidence is rising due to delayed childbearing and a rapid increase in the number of cesarean sections over the last few years. The majority of the patients are either asymptomatic or have mild symptoms and need conservative management only. Myomectomy during cesarean section is routinely avoided due to increased vascularity of the gravid uterus leading to massive hemorrhage, unnecessary obstetric hysterectomy, and increased perioperative morbidity and mortality. However, in modern obstetrics, with advancements in anesthesia, adequate availability of blood products, selective devascularization techniques, and a multidisciplinary approach, obstetricians are increasingly choosing to perform myomectomy during cesarean section, thus saving the patient from future morbidity due to multiple surgeries, anesthetic complications, and out-of-pocket expenditure [2]. Here, we report the case of a successful myomectomy done during an emergency cesarean section without any complications. We intend to break the traditional thumb rule of avoiding myomectomy at the time of cesarean section, and be open to the procedure after careful case selection.

## Case presentation

A 27-year-old gravida 2 para 1 live birth 1 North Indian woman with one previous lower segment caesarean section (LSCS) presented to our outpatient department (OPD) at 35 weeks with complaints of intermittent pain in the lower abdomen that was radiating to her back for the last 5 hours. There was no associated complaint of leaking or bleeding *per vaginum*. Her antenatal period was uneventful. However, she was diagnosed as having a fibroid on the left side of the uterus but reported no complications that could be attributed to it. Her general and systemic examination was unremarkable. All the antenatal investigations were normal. Her recent ultrasound (USG) report suggested a single live intrauterine pregnancy with an intramural fibroid measuring 8.6 × 6.5 cm located in the left anterolateral wall of the lower uterine segment.

**Fig. 1:**
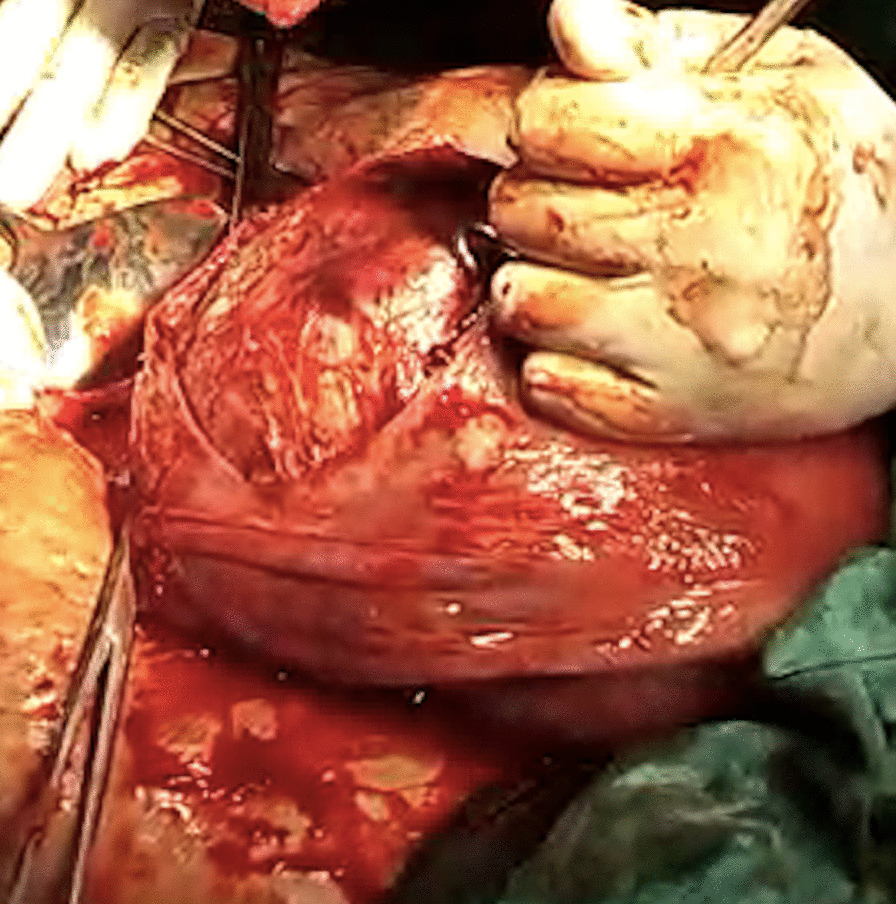
Intraoperative image depicting the fibroid bulging into the incision line

On admission, her heart rate was 96 beats per minute, blood pressure was 110/70 mmHg, and mild pallor was present. Abdominal examination revealed a term size uterus with longitudinal lie. Mild uterine contractions were present with positive scar tenderness. On auscultation, fetal heart rate was 142 beats/minute. Vaginal examination depicted a 2 cm dilated cervix, which was 20–30% effaced, presenting part at −3 station with intact membranes. She was taken up for emergency LSCS given previous cesarean section with preterm labor and scar tenderness. Her preoperative hemoglobin was 12.1 gm%, hematocrit was 34.5%, and blood group was O positive. Adequate blood products were arranged and informed written consent was obtained from the patient and her relatives after explaining to them about the risk of excessive bleeding, need for blood transfusion, and peripartum hysterectomy. During surgery, the abdomen was opened by an infra-umbilical vertical incision for adequate access. There was a single large intramural fibroid occupying most of the lower uterine segment. The previous scar was intact but thinned out, possibly because of the stretching effect of the fibroid. A lower segment transverse incision was made below the inferior margin of the fibroid, and a 2.54 kg female baby was delivered, with an APGAR score of 9 at 1 minute. As the fibroid was bulging into the incision line and causing difficulty in closure of the uterine wound, the decision of myomectomy was taken (Fig. 1). Prophylactically, oxytocin infusion, bilateral ligation of uterine arteries, and injection of vasopressin (diluted) was injected to decrease the blood loss. The fibroid was then enucleated and the myoma bed closed with delayed absorbable sutures followed by closure of the uterine wound. A complete hemostasis was achieved. The total duration of the surgery was approximately 50 minutes and the amount of blood lost around 1100 mL, which is almost comparable to other cesarean sections. Broad-spectrum antibiotics and analgesics were administered in the postoperative period. Her post-surgery hemoglobin was 11.4 gm% and hematocrit was 33%, thus not requiring any blood transfusion. The patient was discharged on the seventh postoperative day with a normal involuting uterus. On follow-up at 6 weeks, the uterus was completely involuted, and repeat USG did not show any fibroid. On further follow-up to 6 months, she was asymptomatic and had an uneventful course.

## Discussion

We report a case of successful myomectomy performed at the time of an emergency cesarean section, with an intent to disintegrate the long-established belief of avoiding it due to fear of complications. Pregnancy with fibroid is a high-risk situation. Although the majority of such cases are asymptomatic or have mild symptoms, 10–40% of cases can present with antenatal complications in the form of pregnancy loss, degenerative changes, malpresentation, abruption placenta, preterm labor, dysfunctional labor or uterine inertia, and increased chances of operative delivery, thus increasing maternal and fetal morbidity and mortality [3]. Treatment is usually conservative during the antenatal period in the form of bed rest, adequate hydration, and analgesics. Myomectomy is rarely required in the case of intractable abdominal pain due to twisting of pedunculated sub-serosal fibroid, red degeneration unresponsive to conservative treatment, or massively enlarged myoma causing abdominal discomfort to the patient [1]. In a recent study, a successful myomectomy was performed during the first trimester at 11 weeks for a large myoma of 14 cm that was a cause of severe discomfort to the patient [4]. The patient continued with pregnancy to term and delivered a healthy baby. Another uneventful myomectomy was performed in the second trimester by Bhatla *et al*. without any adverse impact on pregnancy [5]. Myomectomy during cesarean section is still a topic of debate in the modern era. Until the last decade, it was considered a dreadful surgery except for pedunculated sub-serosal fibroids. However, many researches have concluded that the procedure is not dangerous and does not lead to complications in the hands of an experienced obstetrician [6]. Kwawukume performed cesarean myomectomies on 12 patients and reported that enucleation was much easier in pregnancy due to increased softness of the tissue [7]. A retrospective case–control study, comparing 40 women with fibroids who underwent cesarean myomectomy with 80 women with fibroids forming the control group who underwent cesarean section alone, reported no significant difference in the incidence of hemorrhage between the two groups (12.5% and 11.3%, respectively) [8]. Similar findings were reported in another study, with no significant differences in hemoglobin levels, incidence of blood transfusions, or postoperative pyrexia. However, not all myomas need to be removed, but only those causing difficulty in delivery of the fetus or wound closure and sub-serosal fibroids. In our case, myomectomy was inevitable as the myoma was in the incision line, making wound closure impossible. Every possible effort should be made to reduce the blood loss. Bilateral ligation of uterine arteries immediately after delivery of the fetus significantly reduces both intraoperative and postoperative blood loss and risk of peripartum hysterectomy [9]. It also reduces the recurrence of myomas and minimizes the need for future surgery, with no apparent effect on fertility [10]. This was a key step in our case which prevented the dreaded complications. Also, the postpartum uterus is better adapted physiologically to control bleeding than in any other phase of a woman’s lifetime. The patient and relatives should be properly counseled and informed that removal of myoma is possible, and a final decision can be taken at the time of cesarean based upon the size, number, and location of the fibroid.

## Conclusion

The idea of performing myomectomy at the time of cesarean section appears winsome in a low-resource country like India, where fibroids are common. If performed safely, it can avoid the additional morbidity of a future surgery, thus justifying the cost-effectiveness of the procedure. However, the importance of an expert obstetrician, equipped center with adequate manpower and blood products, and careful case selection cannot be ignored.

## Data Availability

Not applicable.
